# Unveiling the Uncommon: Cushing’s Syndrome (CS) Masquerading as Severe Hypokalemia

**DOI:** 10.7759/cureus.58695

**Published:** 2024-04-21

**Authors:** Warisha Khan, Muzhda Kazman, Huzaifa Abeer, Ali Aizad Raza, Arshan A Khan

**Affiliations:** 1 Critical Care Unit, Basildon University Hospital, Basildon, GBR; 2 Pediatrics, Kabul University of Medical Sciences, Kabul, AFG; 3 Internal Medicine, Mayo Hospital, Lahore, PAK; 4 Pediatrics, Maidstone and Tunbridge Wells Hospital, Royal Tunbridge Wells, GBR; 5 Internal Medicine, Ascension St. John Hospital, Grosse Pointe, USA

**Keywords:** cushing's disease, cushing's syndrome severe hypokalemia, cushing's syndrome hypertension, cushing's syndrome, dexamethasone suppression test

## Abstract

Cushing's syndrome (CS) arises from an excess of endogenous or exogenous cortisol, with Cushing's disease specifically implicating a pituitary adenoma and exaggerated adrenocorticotropic hormone (ACTH) production. Typically, Cushing's disease presents with characteristic symptoms such as weight gain, central obesity, moon face, and buffalo hump.

This case report presents an unusual manifestation of CS in a 48-year-old male with a history of hypertension, where severe hypokalemia was the primary presentation. Initial complaints included bilateral leg swelling, muscle weakness, occasional shortness of breath, and a general feeling of not feeling well. Subsequent investigations revealed hypokalemia, metabolic alkalosis, and an abnormal response to dexamethasone suppression, raising concerns about hypercortisolism. Further tests, including 24-hour urinary free cortisol and ACTH testing, confirmed significant elevations. Brain magnetic resonance imaging (MRI) identified a pituitary macroadenoma, necessitating neurosurgical intervention.

This case underscores the rarity of CS presenting with severe hypokalemia, highlighting the diagnostic challenges and the crucial role of a collaborative approach in managing such intricate cases.

## Introduction

Cushing's syndrome (CS), characterized by excessive cortisol production, is well-known for its diverse and often conspicuous clinical manifestations. Cushing's disease is a subset of CS resulting from a pituitary adenoma overproducing adrenocorticotropic hormone (ACTH), leading to heightened cortisol secretion. The classic presentation involves a spectrum of symptoms such as weight gain, central obesity, muscle weakness, and mood alterations [1].

Despite its classic presentation, CS can demonstrate diverse and atypical features, challenging conventional diagnostic paradigms. This case report sheds light on a rare manifestation of CS, where severe hypokalemia was the primary clinical indicator. Notably, instances of CS prominently manifesting through severe hypokalemia are scarce in the literature [1,2].

Through this exploration, we aim to provide valuable insights into the diagnostic intricacies of atypical CS presentations, underscore the significance of a comprehensive workup, and emphasize the collaborative approach essential for managing such uncommon hormonal disorders.

## Case presentation

A 48-year-old male with a history of hypertension presented to his primary care physician with complaints of bilateral leg swelling, occasional shortness of breath, dizziness, and a general feeling of malaise persisting for 10 days. The patient reported increased water intake and urinary frequency without dysuria. The patient was diagnosed with hypertension eight months ago. He experienced progressive muscle weakness over two months, hindering his ability to perform daily activities, including using the bathroom. The primary care physician initiated a blood workup that revealed severe hypokalemia with a potassium level of 1.3 mmol/L (reference range: 3.6 to 5.2 mmol/L), prompting referral to the hospital.

Upon admission, the patient was hypertensive with a blood pressure of 180/103 mmHg, a heart rate of 71 beats/minute, a respiratory rate of 18 breaths/minute, and an oxygen saturation of 96% on room air. Physical examination revealed fine tremors, bilateral 2+ pitting edema in the lower extremities up to mid-shin, abdominal distension with normal bowel sounds, and bilateral reduced air entry in the bases of the lungs on auscultation. The blood work showed the following findings (Table 1).

**Table 1:** Blood work findings

Parameter	Result	Reference Range
Potassium (K)	1.8 mmol/L	3.5-5.0 mmol/L
Sodium (Na)	144 mmol/L	135-145 mmol/L
Magnesium (Mg)	1.3 mg/dL	1.7-2.2 mg/dL
Hemoglobin (Hb)	15.5 g/dL	13.8-17.2 g/dL
White blood cell count (WBC)	13,000 x 10^3^/µL	4.5 to 11.0 × 10^9^/L
Platelets	131,000 x 10^9^/L	150-450 x 10^9^/L
pH	7.57	7.35-7.45
Bicarbonate (HCO_3_)	46 mmol/L	22-26 mmol/L
Lactic acid	4.2 mmol/L	0.5-2.0 mmol/L

**Figure 1:**
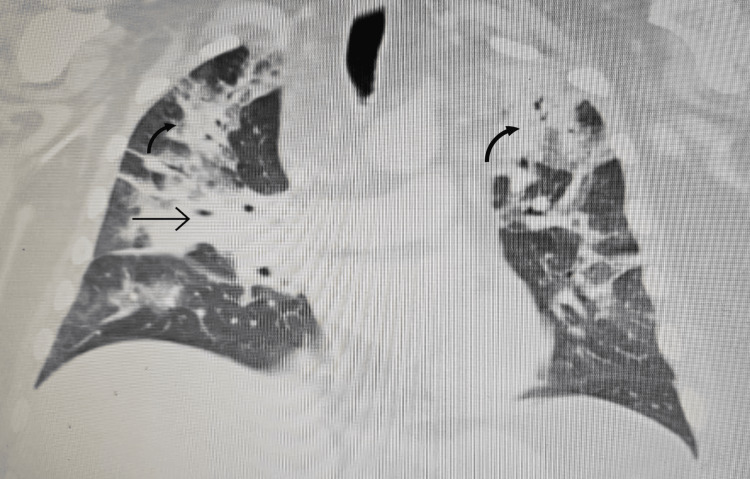
A chest X-ray revealing (arrows) bilateral multilobar pneumonia

In order to correct the electrolyte imbalances, the patient received intravenous (IV) magnesium and potassium replacement and was later transitioned to oral. The patient was also started on normal saline at 100 cc per hour. To further investigate the complaint of shortness of breath, the patient underwent a chest X-ray, which revealed bilateral multilobar pneumonia (Figure 1). He was subsequently treated with ceftriaxone (1 g IV daily) and clarithromycin (500 mg twice daily) for seven days.

**Figure 2:**
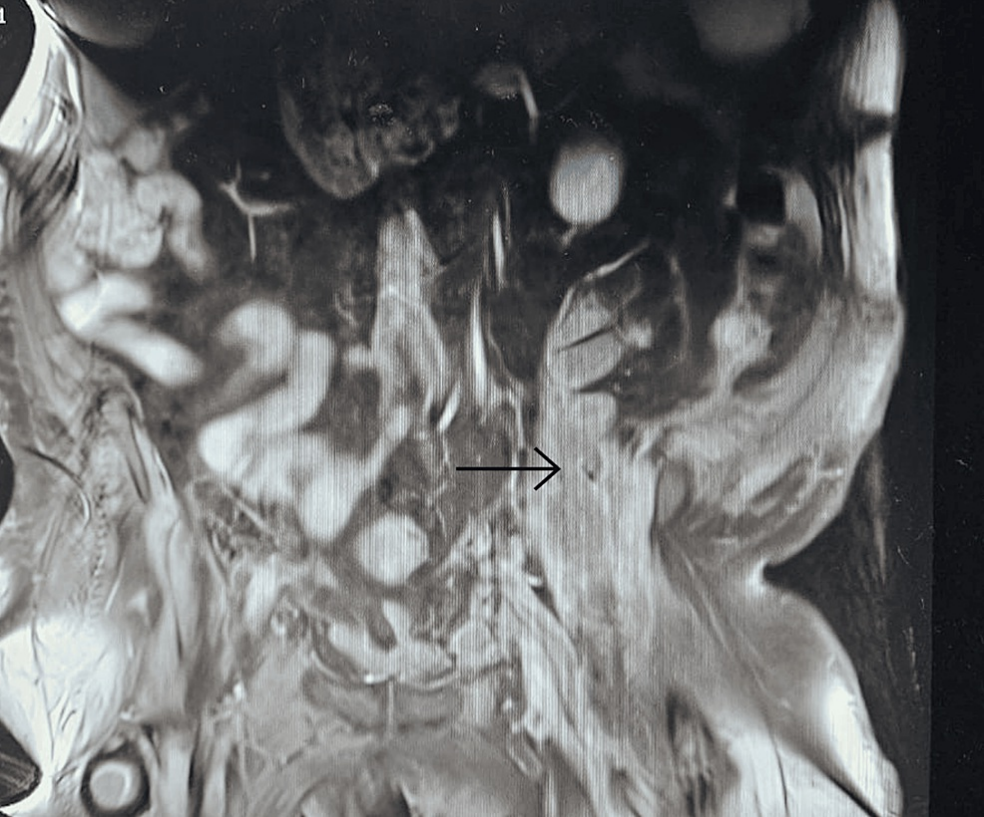
Magnetic resonance imaging (MRI) depicting an 8x8 cm hematoma (arrow) involving the left psoas and iliacus muscles

With persistent abdominal pain and lactic acidosis, a computed tomography (CT) scan abdomen and pelvis with contrast was conducted, revealing a psoas muscle hematoma. Subsequent magnetic resonance imaging (MRI) depicted an 8x8 cm hematoma involving the left psoas and iliacus muscles. The interventional radiologist performed drainage of the hematoma involving the left psoas and iliacus muscles (Figure 2).

In light of the concurrent presence of hypokalemia, hypertension, and metabolic alkalosis, there arose concerns about Conn's syndrome, prompting consultation with endocrinology. Their recommended workup for Conn's syndrome included assessments of the aldosterone-renin ratio and random cortisol levels. The results unveiled an aldosterone level below 60 pmol/L (reference range: 190 to 830 pmol/L in SI units) and a plasma renin level of 0.2 pmol/L (reference range: 0.7 to 3.3 mcg/L/hr in SI units). Notably, the aldosterone-renin ratio was low, conclusively ruling out Conn's syndrome. The random cortisol level was notably elevated at 1334 nmol/L (reference range: 140 to 690 nmol/L).

Furthermore, a low-dose dexamethasone suppression test was undertaken due to the high cortisol levels. Following the administration of 1 mg of dexamethasone at 10 p.m., cortisol levels were measured at 9 p.m., 3 a.m., and 9 a.m. the following day. The results unveiled a persistently elevated cortisol level surpassing 1655 nmol/L, signaling an abnormal response to dexamethasone suppression and raising concerns about a hypercortisolism disorder, such as CS.

In the intricate progression of this case, the investigation delved deeper with a 24-hour urinary free cortisol level, revealing a significant elevation at 521 mcg/day (reference range: 10 to 55 mcg/day). Subsequent testing of ACTH portrayed a markedly elevated level of 445 ng/L, distinctly exceeding the normal reference range of 7.2 to 63.3 ng/L. A high-dose 8 mg dexamethasone test was performed to ascertain the source of excess ACTH production. The baseline serum cortisol levels before the high-dose dexamethasone suppression test were 1404 nmol/L, which decreased to 612 nmol/L afterward, strongly suggesting the source of excess ACTH production to be in the pituitary gland.

**Figure 3:**
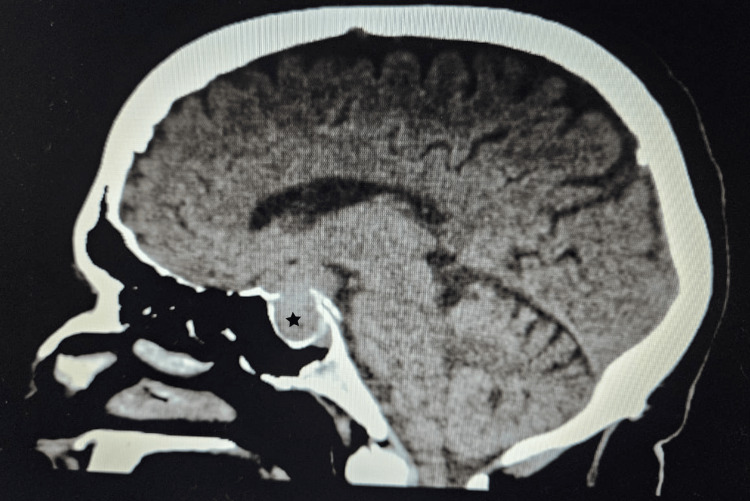
Magnetic resonance imaging (MRI) of the brain depicting a 1.3x1.3x3.2 cm pituitary macroadenoma (star)

A CT scan of the adrenal glands ruled out adrenal mass, while an MRI of the brain uncovered a 1.3x1.3x3.2 cm pituitary macroadenoma (Figure 3), leading to compression of adjacent structures. Neurosurgery was consulted, and they recommended surgical removal of the macroadenoma due to the tumor size and potential complications. The patient was referred to a tertiary care hospital for pituitary adenoma removal.

## Discussion

CS represents a complex endocrine disorder characterized by excessive cortisol production. While the classic presentation of CS includes weight gain, central obesity, and muscle weakness, our case highlights an uncommon initial manifestation: severe hypokalemia. This atypical presentation underscores the diverse clinical spectrum of CS and the challenges it poses in diagnosis and management [1,2].

While CS typically presents with the classic symptoms mentioned above, severe hypokalemia as the initial manifestation is exceedingly rare. Hypokalemia in CS often results from excess cortisol-mediated activation of mineralocorticoid receptors, leading to increased urinary potassium excretion and renal potassium wasting. Additionally, metabolic alkalosis secondary to cortisol excess further exacerbates hypokalemia [3,4].

Diagnosing a case of Cushing's disease typically commences with a thorough examination of the patient's medical history and a comprehensive physical assessment aimed at identifying characteristic manifestations such as central obesity, facial rounding, proximal muscle weakness, and increased susceptibility to bruising. Essential to confirming the diagnosis are laboratory examinations, which involve measuring cortisol levels through various tests, including 24-hour urinary free cortisol testing, late-night salivary cortisol testing, and dexamethasone suppression tests. Furthermore, assessing plasma ACTH levels aids in distinguishing between pituitary-dependent and non-pituitary causes of CS. Integral to the diagnostic process are imaging modalities such as MRI of the pituitary gland, which facilitate the visualization of adenomas and the determination of their size and precise location [1-4].

Treatment for Cushing's disease primarily entails surgical removal of the pituitary adenoma via transsphenoidal surgery, with the aim of excising the tumor and restoring normal pituitary function. In cases where surgical intervention is unsuitable or unsuccessful, pharmacological therapies employing medications such as cabergoline (a dopamine receptor agonist) or pasireotide (a somatostatin analogue) may be considered to suppress ACTH secretion and regulate cortisol levels. Additionally, radiation therapy, whether conventional or stereotactic radiosurgery, serves as a supplementary or alternative treatment approach to reduce tumor dimensions and mitigate ACTH production [5,6]. To assess the effectiveness of treatment, manage any problem, and assure long-term illness remission, diligent long-term follow-up and monitoring are essential. Collaborative multidisciplinary care involving specialists such as endocrinologists, neurosurgeons, and other healthcare professionals is pivotal in optimizing patient outcomes and enhancing overall quality of life [2,4].

The prognosis of CS largely depends on the underlying cause, stage of the disease, and efficacy of treatment. Early recognition and prompt intervention are essential for improving outcomes and minimizing long-term complications. Surgical resection of the adrenal or pituitary tumor can lead to remission of CS in the majority of cases. However, recurrence rates vary depending on factors such as tumor size, invasiveness, and completeness of resection [2,3]. Long-term follow-up with endocrinologists is crucial for monitoring disease recurrence, assessing hormonal function, and managing comorbidities associated with CS.

## Conclusions

In conclusion, our case report highlights the rarity of severe hypokalemia as the initial presentation of CS. This unique presentation underscores the diverse clinical manifestations of CS and emphasizes the diagnostic challenges encountered in clinical practice. A multidisciplinary approach involving endocrinologists, neurosurgeons, and radiologists is essential for the timely diagnosis and management of CS. Early recognition, prompt intervention, and long-term follow-up are essential for optimizing outcomes and improving the quality of life for patients with this endocrine disorder.
